# Human Myometrial and Uterine Fibroid Stem Cell-Derived Organoids for Intervening the Pathophysiology of Uterine Fibroid

**DOI:** 10.1007/s43032-022-00960-9

**Published:** 2022-05-18

**Authors:** Saswati Banerjee, Wei Xu, Indrajit Chowdhury, Adel Driss, Mohamed Ali, Qiwei Yang, Ayman Al-Hendy, Winston E. Thompson

**Affiliations:** 1 Department of Physiology, Morehouse School of Medicine, 720 Westview Drive Southwest, Atlanta, GA 30310 USA; 2 Department of Obstetrics and Gynecology, Morehouse School of Medicine, Atlanta, GA USA; 3 Clinical Pharmacy Department, Ain Shams University, Cairo, Egypt; 4 Department of Obstetrics and Gynecology, University of Chicago, Chicago, IL USA

**Keywords:** Uterine fibroids, Estrogen, Progesterone, Organoid

## Abstract

Uterine fibroids (UFs) (leiomyomas or myomas) are the most common clonal neoplasms of the uterus in women of reproductive age worldwide. UFs originate from myometrium consist of smooth muscle and fibroblast components, in addition to a substantial amount of fibrous extracellular matrix which all contribute to the pathogenetic process. Current treatments are primarily limited to surgical and interventional. Here, we have established a novel and promising organoid model from both normal and patient myometrial stem cells (MMSCs). MMSCs embedded in Matrigel in stem cell media swiftly formed organoids which successfully proliferate and self-organized into complex structures developing a sustainable organoid culture that maintain their capacity to differentiate into the different cell types recapitulating their tissue of origin and shows responsiveness to the reproductive hormones (estrogen and progesterone). Gene expression analysis and structural features indicated the early onset of uterine fibrosis led to the accumulation of extracellular matrix suggesting the potential use of this model in better understanding of the pathophysiology associated with UFs and inventing novel therapeutics for the treatment of UFs.

## Introduction

Uterine fibroids (UFs; leiomyomas) are benign monoclonal neoplasms of the myometrium (MM) and represent the most common tumors in women worldwide [1–4]. The collective incidence of UFs is manifested to exceed 77% in premenopausal women by the age of 45. These benign smooth muscle tumors are associated with the excessive deposition of the extracellular matrix (ECM) that plays a major role in the enlargement and stiffness of these tumors. UFs contribute to gynecologic and reproductive dysfunction, ranging from menorrhagia and pelvic pain to infertility, recurrent miscarriage, and pre-term labor are nonetheless associated with significant morbidity [5]. The traditional treatments include hysterectomy or myomectomy, and hormonal agents that contribute substantially to a healthcare cost of up to $34 billion dollars each year [6]. Remarkably, while race and environmental exposures have been clearly shown to increase the risk for this disease, neither the determinants nor biomarkers of risk nor the targets for environmental exposures have not yet been defined, which could provide a foundation for the design of interventions to prevent the development or ameliorate the impact of these tumors.

The prevailing model for UF pathogenesis invokes the genetic transformation of a single myometrial stem cell (MMSC) into a tumor-initiating cell that seeds and sustains clonal tumor growth, characterized by an increase in cell size and number and abundant extracellular matrix production, under the influence of endocrine, autocrine, and paracrine growth factors and hormone receptor signaling [7]. Most importantly, the fibroid stem cells acquire the mutations in the gene encoding Mediator complex subunit *MED12* and are dominant drivers of UFs in women of diverse racial and ethnic origins [8]. It has been also established that human Stro-1/CD44 positive myometrial and fibroid cells can differentiate into mesenchymal lineage cell types, and finally form myometrial-/fibroid-like-tissues in an animal model [9, 10]. The other risk factors underlying the pathogenesis of UF have been identified as environmental exposures and modifiable lifestyle factors. The genome-wide association studies and analyses of UF tumors have revealed several genetic and epigenetic factors that are correlated with an increased risk for UF [11].

Despite the growing knowledge of UF pathogenesis the prevalence of UFs with limited treatment options warrant better models to validate the impact of genetic transformation of stem cells as well as the developmental exposures. The costly process of validation in animal models and the difference in the mechanism of metabolism and toxicology between species have a significant restriction on determining the mechanism and drug discovery for UFs. Moreover, the traditional 2D cell culture does not mimic the in vivo environment because they lose their phenotype quickly. Over the past decade, a 3D multicellular in vitro tissue construct named organoid has been developed that mimics its corresponding in vivo organ, such that it can be used to study aspects of that organ in the tissue culture dish [12]. Organoids from various human epithelial tissues are rapidly being developed for in vitro studies and offer several advantages over monolayer cultures and cell lines [13–17]. They contain both progenitor stem cells and differentiated cells in a self-organizing genetically stable 3D system that closely resembles the tissue of origin. Chemically defined conditions maintain sufficient stemness to allow long-term culture while differentiation can be induced by altering signaling via exogenous factors. As such, they can be employed as a readily available substitute for freshly isolated primary cells. So far, little is known regarding the three-dimensional in vitro model in UFs, and surprisingly, no studies have heretofore attempted to use MMSCs, the origin of the cell for UF to develop the in vitro model and bridge this significant knowledge and technology gap.

Recent efforts to establish 3D uterine epithelial cultures have been successful as regards of forming hormone responsive epithelial organoids. Here, we for the first time developed functional myometrial organoids both from normal and patient MMSCs. A striking feature of uterine fibroids is their dependency on ovarian steroids, estrogen, and progesterone [18]. Ovarian activity is essential for fibroid growth, and most fibroids shrink after menopause. The sharp elevations and declines in the production of estrogen and progesterone that are associated with very early pregnancy and the postpartum period have a dramatic effect on fibroid growth [19, 20].

Current study shows that the MMSCs and UFSCs maintain the stemness in 2D culture under normoxic conditions which successfully proliferate and self-organized into complex structures developing a sustainable organoid culture that maintains their capacity to differentiate into the different cell types of their tissue of origin and shows responsiveness to the reproductive hormones (estrogen and progesterone).

## Materials and Methods

### Isolation and Purification of Myometrium Stem Cells from Human Uterine Fibroids

The normal myometrial and fibroid stem cells were isolated using a method as described previously [10]. Samples of human myometrium and fibroids were collected from women undergoing hysterectomy or myomectomy for symptomatic uterine fibroids, (age range: 30–60) excluding other gynecological disorders or malignancies. Stro-1/CD44 were used as specific surface markers to enrich a subpopulation of myometrial/fibroids cells, exhibiting key features of stem/progenitor cells [10].

### Growth, Maintenance, and Characterization of Myometrial Stem Cells

MMSCs were grown and routinely maintained in DMEM and Ham’s F12 (Fisher Scientific, Waltham, MA) supplemented with 12% FBS (Fisher Scientific, Waltham, MA) and 1% antibiotic–antimycotic (Fisher Scientific, Waltham, MA) mixture. Once confluent, cells were plated on a 4-well chamber slide for immunocytochemical analysis, or cell pellet was collected for gene expression analysis by real-time PCR.

### Formation, Maintenance, and Hormonal Treatment of Human Myometrial Organoids

Once confluent, the MMSCs were dissociated with 0.25% Trypsin and 0.1% EDTA in HBSS w/o calcium, magnesium (Fisher Scientific, Waltham, MA), and the pellet was collected by centrifugation. The supernatant was removed; the cell pellet was suspended in serum-free MesenCult™-ACF Plus Medium (Stemcell Technologies, Vancouver, Canada) and pipetted up and down to break any clumps and kept on ice. Cells were counted using a TC20 automated cell counter (Bio-Rad Laboratories, Hercules, CA). About 10,000 cells were mixed with 2 µL of Matrigel (Corning, Corning, NY) in a final volume of 100 µL and added to V-bottom plates (AmsBio, Abingdon, UK) and incubated at a 37 °C incubator with 5% CO_2_. Once the cells form a spheroid structure, each spheroid was transferred to each well of 24-well plates, and pictures of the individual organoids were taken before and after hormonal treatment maintained in serum-free media. In a previous study to examine hormone responsiveness, myometrial tissue cultures were incubated with physiological concentrations of progesterone (P4; 500 nM) and/or estradiol (E2; 400 nM) which are equivalent to 157.23 ng/ml and 108 ng/ml respectively [21]. We did a dose–response study with 10 ng/mL, 100 ng/mL, and 500 ng/mL of either estrogen or progesterone; and 10nG/mL was selected due to the toxic effects at higher dosages (data not shown). After 7 days, organoids were treated with either 10 ng/ml of either estrogen (E2) or progesterone (P4) or vehicle as control (Sigma-Aldrich, St. Louis, MO) for 72 h. The pictures of the organoids were taken before and after treatment for area analysis using an Olympus BX41 microscope (Olympus America, Center Valley, PA). To analyze the comprehensive in situ assessment on treatment responses in 3D cultures, Image J software was used on the microscopic images to measure the area of the organoids. Also, organoids were harvested and collected for immunohistochemical, RNA, and protein analysis.

### Immunocytochemistry

The MMSCs were plated in 4-well chamber slides in the culture media as mentioned earlier. Next day, the cells were washed with PBS and fixed with 4% paraformaldehyde for 15 min and permeabilized with cold methanol for 5 min. After washings, cells were blocked with 5% FBS in PBS containing 0.2% Triton x-100 PBST and incubated overnight with primary antibodies (Table 1) in the blocking buffer. The cells were washed in PBST three times and then incubated for 1 h in the dark in Alexa-Fluor secondary antibodies, 1:1000 (Life Technologies, Carlsbad, CA). After washing with PBST twice, the cells were incubated with DAPI (500 ng/mL, Sigma-Aldrich, St. Louis, MO). Images were acquired using Olympus BX41 microscope (Olympus America, Center Valley, PA). The antibodies used in this study are listed in Table 1.

**Table 1:** Antibodies for immunohistochemical and cytochemical staining

Antigen	RRID	Species reactivity	Host	Company	Dilution used
Cd44	AB_2806740	Human, mouse, rat	Rabbit	Invitrogen	1:250
Stro1	AB_628298	Human	Mouse	Santa Cruz	1:200
Vimentin	AB_628437	Human, mouse, rat	Mouse	Santa Cruz	1:200
Collagen type I	AB_731684	Mouse, rat, sheep, goat, horse, cow, human, pig, common marmoset	Rabbit	enQuire Bioreagent	1:100
Fibronectin	AB_2262874	Human, mouse	Rabbit	Abcam	1:100
POSTN	AB_1854827	Human	Rabbit	Sigma	1:200
Smooth muscle actin	AB_2572996	Human, mouse, rat	Mouse	Invitrogen	1:500
Smooth muscle actin	AB_10003706	Hu, Mu, Rt, Ca, Ze, Po, Bv, Ch, Rb, RM	Rabbit	Novus Biologicals	1:250
Estrogen receptor α	AB_2632959	Hu	Rabbit	Cell signaling	1:200
Progesterone receptor A/B	AB_2797144	Hu	Rabbit	Cell signaling	1:200

To embed the organoids for cryosection, the organoids were transferred to 1.5-mL tubes and centrifuged, and the pellets were washed with PBS. The organoids were then fixed with 4% paraformaldehyde for 20 min and washed with PBS. The pelleted organoids were then resuspended in 2% methylene blue for staining, washed with PBS, and cryoprotected overnight by suspending in 30% sucrose. Next day, the sucrose was removed by centrifugation, and organoids were embedded in OCT (Fisher Scientific, Waltham, MA) and flash-frozen at − 80 °C. The embedded organoids in OCT were sectioned using a Cryostat (Thermo Scientific™ Microm™ HM 550, Fisher Scientific, Waltham, MA). The sections were preserved at 4 °C for staining. The staining was done following the method described earlier. The antibodies used are listed in Table 1.

### Quantitative PCR

Total RNA was extracted using Qiazol Reagent (Qiagen, Valencia, CA) according to the manufacturer’s instructions. Reverse transcription was performed using iScript cDNA synthesis kit (Bio-Rad Laboratories, Hercules, CA). QPCR was carried out using SsoAdvanced Universal SYBR Green Supermix (Bio-Rad Laboratories, Hercules, CA) on CFX Connect Real-Time PCR Detection System (Bio-Rad Laboratories, Hercules, CA). The relative expression of gene was calculated using 2^−ΔΔCT^ method and 18 s ribosomal RNA as the reference gene. All qPCR data are presented as mean ± SEM of biological triplicates. Statistical analysis was performed using unpaired *t* test results. The two-tailed *p* values less than 0.05 were statistically significant. The sequence information for all PCR primers can be found in Table 2.

**Table 2:** Primers used for real-time PCR

Name of the gene	Forward primer	Reverse primer	References
Rad50	GCGGAGTTTTGGAATAGAGGAC	GAGCAACCTTGGGATCGTGT	[22]
Rad51	TCTCTGGCAGTGATGTCCTGGA	TAAAGGGCGGTGGCACTGTCTA	[23]
Chk1	CCCGCACAGGTCTTTCCTT	GGCTGGGAAAAGCTGATCC	[24]
chk2	ATGTCTCGGGAGTCGGATGT	TCACAACACAGCAGCACACA	[25]
ATM	CTC TGA GTG GCA GCT GGA AGA	TTT AGG CTG GGA TTG TTC GCT	[26]
MRE1	TCAGCATCGAGAGGAGGGTCT	GACATTTCGGGAAGGCTGCT	[27]

### Statistical Analysis

All experimental data are expressed as mean ± standard error of the mean of five independent experiments. Statistical analysis was carried out by unpaired *t* test.

## Results

### Human Myometrial/Fibroid Cd44/Stro1 Stem Cells Differentiated Under Normoxic Conditions with a Downregulation of DNA Repair Effector Molecules in Fibroid Stem Cells

**Fig. 1:**
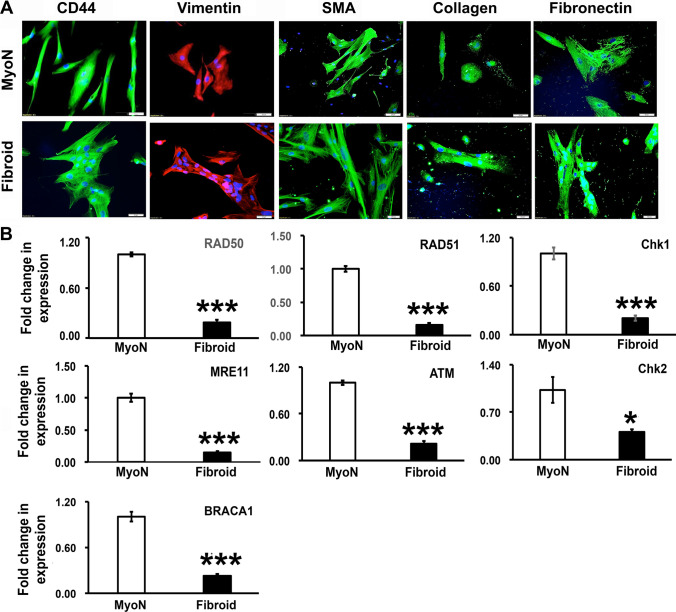
Uterine fibroid (UF) stem cells exhibit reduced expression of DNA repair effectors. CD44/Stro1 +  + stem cells (MMSCs) were isolated from human normal myometrial (MyoN) and uterine fibroid (UF) biopsies using enzymatic digestion and flow cytometry. The stemness of the myometrial stem cells (MSCs) from MyoN and UF were analyzed in 2D culture under normoxic condition in MesenCult™-ACF Plus Medium. **A** Representative immunofluorescence images showing the localization of the stem cell marker CD44 (green), the stromal cell marker vimentin (red), the smooth muscle cell marker actin (green), and extracellular matrix proteins marker collagen (green) and fibronectin (green) in MyoN and UF stem cell. The nuclei are counter stained in 4′,6-diamidino-2-phenylindole (DAPI, blue). Scale bar = 50 µM. **B** Real-time PCR analysis of the expression of DNA-double strand breaks (DSB) repair-related genes including RAD50, RAD51, CHK1, CHK2, ATM, and MRE11 in MMSCs derived from UF tissue compared to MyoN. The relative changes in gene expression were quantified by 2 − ΔΔCT method, and the ∆∆Ct was calculated using the average of the control values. All the data and numerical values are presented as mean + / − standard error of the mean from five independent experiments performed for each individual group

Increasing evidence indicates that putative myometrial stem/progenitor cells may contribute to both physiologic and pathologic processes underlying the formation of UFs, and hence identification of the specific surface markers characterizing these cells remains critically important. Previous studies have established that several individual and combinations of stem/progenitor cell markers including Oct4 [28], CD34/CD49f/b [29], CD44/Stro1 [10], and CD140b/CD146 + or SUSD2 [30] were used to identify and characterize those cells from myometrium and UFs. Previously published data also suggest that CD34/CD49 +  + [31] and or Stro11/CD44 +  + [10] cells are comparable and likely to represent the same population of stem cells although isolated using different surface markers [32]. As it is mentioned in the Materials and Methods section, we used the subpopulation of myometrial stem cells with Stro1/CD44 surface markers, enriched from myometrial stem/progenitor-like cells (MSCs) expressing stem cell markers Oct-4/c-kit/nanog [32]. Stro-1/CD44 stem cells were isolated from human myometrial and uterine fibroid biopsies using enzymatic digestion and flow cytometry. We analyzed the stemness of the MMSCs in 2D culture under normoxic conditions in MesenCult™-ACF Plus Medium by immunocytochemistry. The cells consisted of subpopulations of stem cells and differentiated cells in monolayer culture (Fig. 1A). Under normoxic conditions, the cells only retain the expression of CD44 among the dual surface markers (CD44/Stro1) and get differentiated into stromal cells (vimentin) and smooth muscle cells (smooth muscle actin) and produces extracellular matrix markers (collagen-I and fibronectin) (Fig. 1A). The epithelial marker E-cadherin was not expressed (data not shown). Earlier studies have revealed the higher mutational heterogeneity in UFs as compared to normal myometrial cells and that could be attributed to a reduced expression of DNA repair effectors [33]. So, the expression of the DNA repair effectors was measured in MMSCs derived from normal myometrium and fibroid tissues grown under normoxic conditions. RNAs were isolated from confluent normal myometrial and fibroid positive Stro-1/CD44 stem cell culture, and qRT-PCR analysis revealed that there is a significant decrease in the expression of several DNA-double strand breaks (DSB) repair-related genes including *RAD50*, *RAD51*, *CHK1*, *CHK2*, *ATM*, and *MRE11* in UFSCs derived from fibroid tissue compared to MMSCs derived from uterus without UFs (Fig. 1B).

### Derivation of Myometrial Organoids from Cd44/Stro1 Stem Cells

**Fig. 2:**
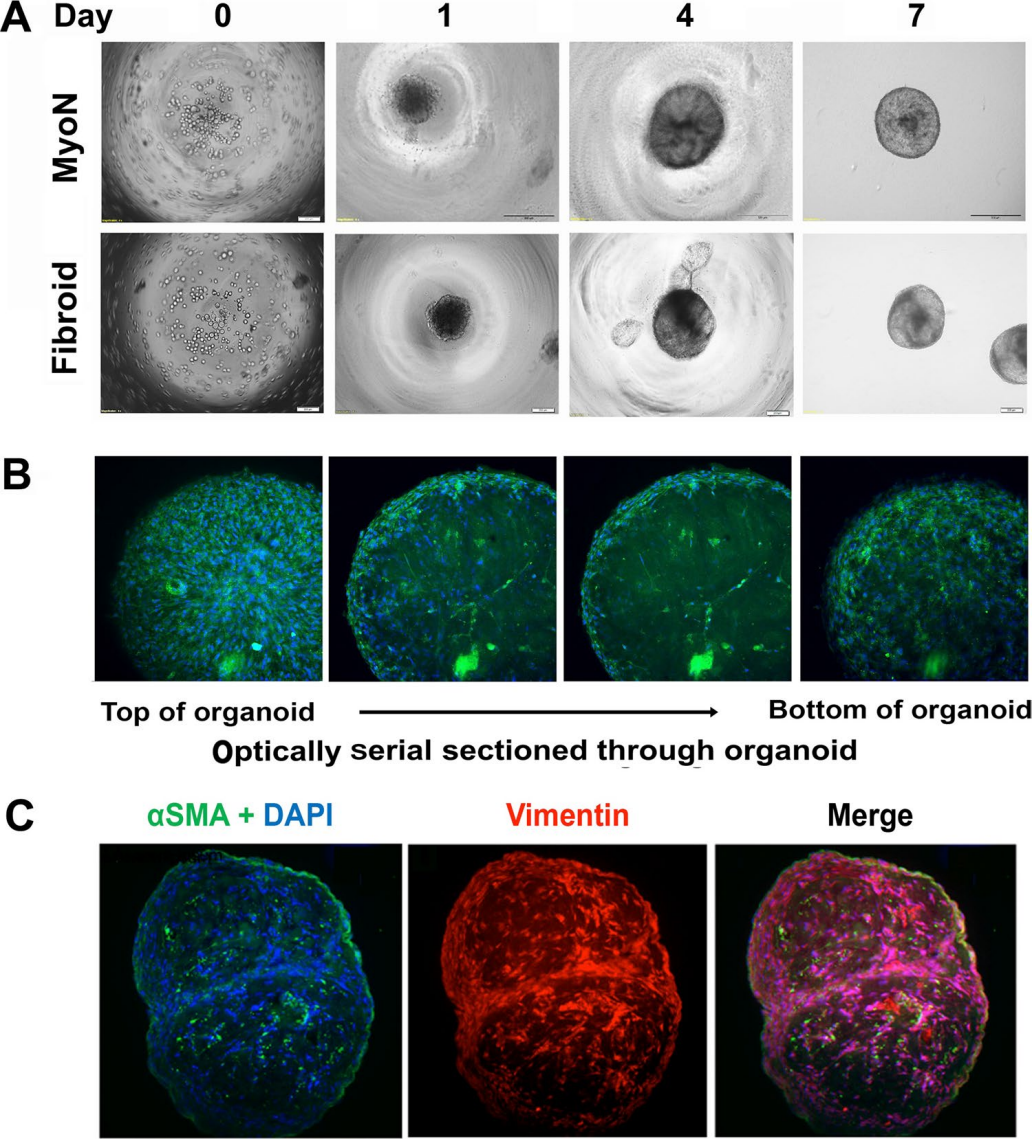
The establishment of in vitro organoid model from human CD44/Stro1 +  + myometrial stem cells and UF stem cells derived from normal myometrial and uterine fibroid (UF) tissue. The human CD44/Stro1 +  + myometrial stem cells (MMSCs) derived from normal myometrial (MyoN) and uterine fibroid (UF) tissue was grown in 2D culture. Upon confluency, ~ 10,000 cells were mixed with 2 µL of Matrigel in 100µL and grown in a V-bottom 96-well plate at 37 °C with 5% CO_2_. On day 6, once the cells formed a spheroid structure, each spheroid was transferred to each well of 24-well plate for further growth (for detail see Material and Methods). **A** Representative phase pictures are demonstrating the development of MyoN and UF organoid from MMSC in vitro. **B** On day 7, whole intact organoids were fixed, and immunofluorescence staining was done with alpha-smooth muscle actin (green). The nuclei are counterstained in 4′,6-diamidino-2-phenylindole (DAPI, blue). Representative serial confocal images are optically sectioned and showing the smooth muscle actin cells which are organized to the exterior of the organoid with few labeled cellular structures within the interior. **C** On day 7, the organoids were cryosectioned, and immunostaining was done with alpha smooth muscle actin (green) and vimentin (red). Representative confocal images are demonstrating the intercalation of SMA with stromal cell marker vimentin. The nuclei are counterstained in 4′,6-diamidino-2-phenylindole (DAPI, blue; all the data are from five independent (*n* = 5) experiments performed for each individual group

We optimized that the MMSCs at a seeding density of 10,000 cells form reproducible organoids under a defined culture system utilizing Matrigel to provide an extracellular matrix for embedded cells and MesenCult™-ACF Plus Medium for 6 days in an ultra-low attachment 96-well plate (Fig. 2A). Under these conditions, cells self-organize by day 1 forming spheroid structures and grow exponentially in size and cell number within 6 days (Fig. 2A). On day 6, well-defined organoids were transferred to 24-well plates (Fig. 2B). On day 7, whole intact organoids were fixed, and immunofluorescence staining was done for alpha smooth muscle actin antibody. As shown in Fig. 2B in the serial confocal images, smooth muscle actin cells were organized to the exterior of the organoid with few labeled cellular structures within the interior. To gain further insight into the cellular structure, the day 7-frozen organoids were sectioned by cryostat (Thermo Scientific™ Microm™ HM 550, Fisher Scientific, Waltham, MA) and stained for alpha smooth muscle actin and vimentin, and images were acquired using 3i Marianas Lightsheet fluorescence microscope (Intelligent Imaging Innovations, Inc., USA), fitted with 10 × illumination objectives and an achromatic 10 × detection objective. At this time point, we observed within the interior of the organoids, smooth muscle cells intercalated with stromal cells (Fig. 2C).

### Uterine Normal Myometrial and Fibroid Organoids Exhibit a Morphological and Physiological Difference in Hormone Responsiveness

**Fig. 3:**
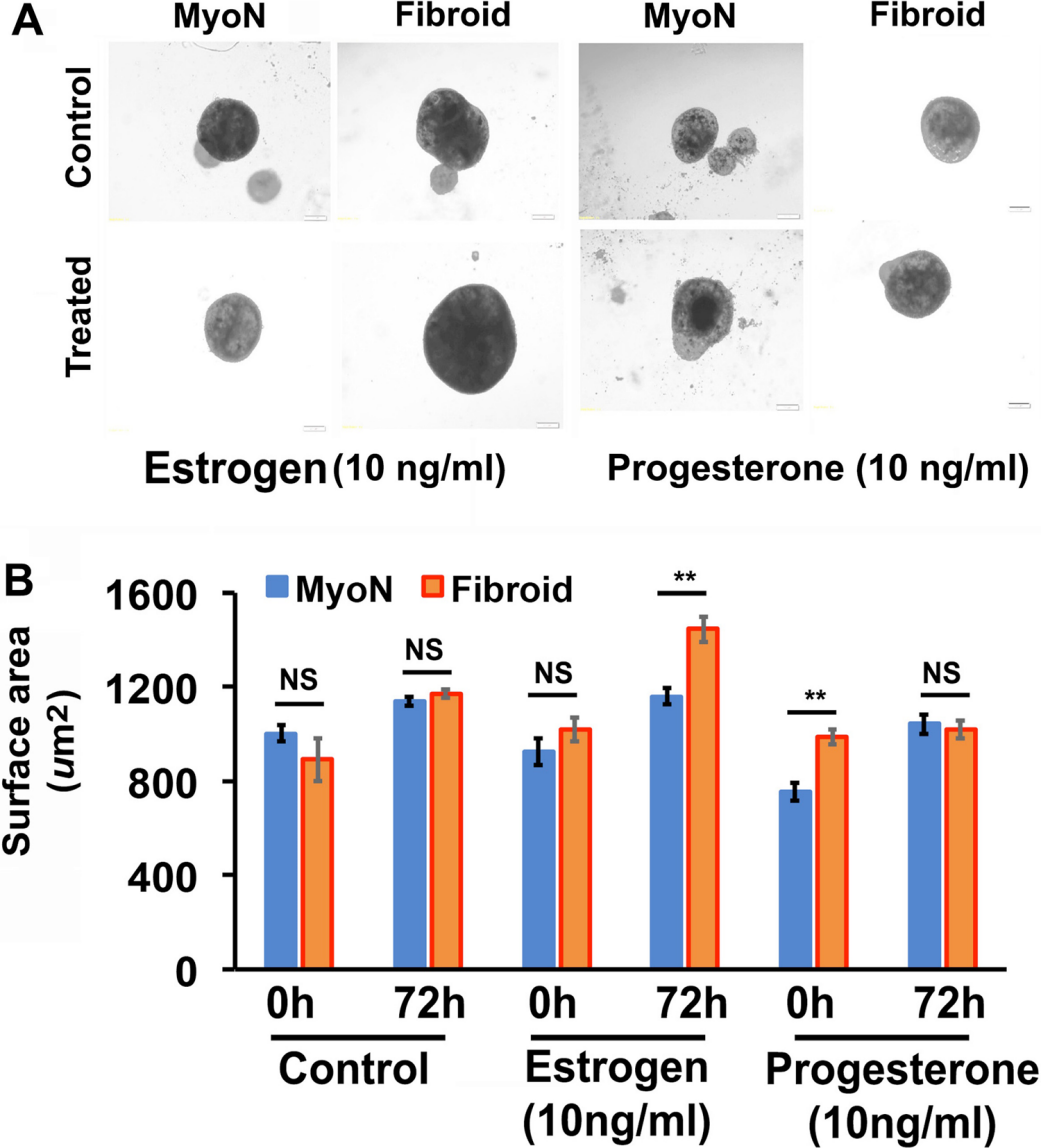
Human myometrial organoids derived from CD44/Stro1 +  + stem cells exhibit a significant response to ovarian steroid hormones in vitro. On day 7, the organoids derived from human MMSC and UFSC were treated with estradiol (E2, 10 ng/ml) or progesterone (P4, 10 ng/ml) for 72 h. **A** Representative phase-contrast microscopy pictures are exhibiting the effects of E2 and P4 on the growth of MMSC-derived MyoN and UF organoids. **B** The bar graph is a quantitative representation of the difference in surface area of the MMSC-derived MyoN and UF organoids in response E2 and P4. Data are from five independent (*n* = 5) experiments performed for each individual group. The bar graphs represent the mean ± SEM of results (*n* = 5). Asterisks (*) represent unpaired Student’s *t* test, *p ≤ 0.01, **p ≤ 0.001, ***p ≤ 0.0001, NS, not significant

The human uterine myometrium is responsive to ovarian steroid hormones. To investigate the hormone responsiveness of organoids in vitro, at the 7th day, the myometrial organoids were treated with 10 ng/ml of estrogen or progesterone for 72 h. After 72 h, marked visible differences in size were observed among the treated and untreated organoids. ImageJ analysis revealed that there is a significant change in the area size of control groups of both MMSC- and UFSC-derived organoids but there is a greater increase in area of UFSC-derived organoids compared to MMSC. Although there is a significant increase in area size in response to estrogen treatment in both MMSC- and UFSC-derived organoids, progesterone does not cause any further increase in UFSC organoids (Fig. 3A and B).

**Fig. 4:**
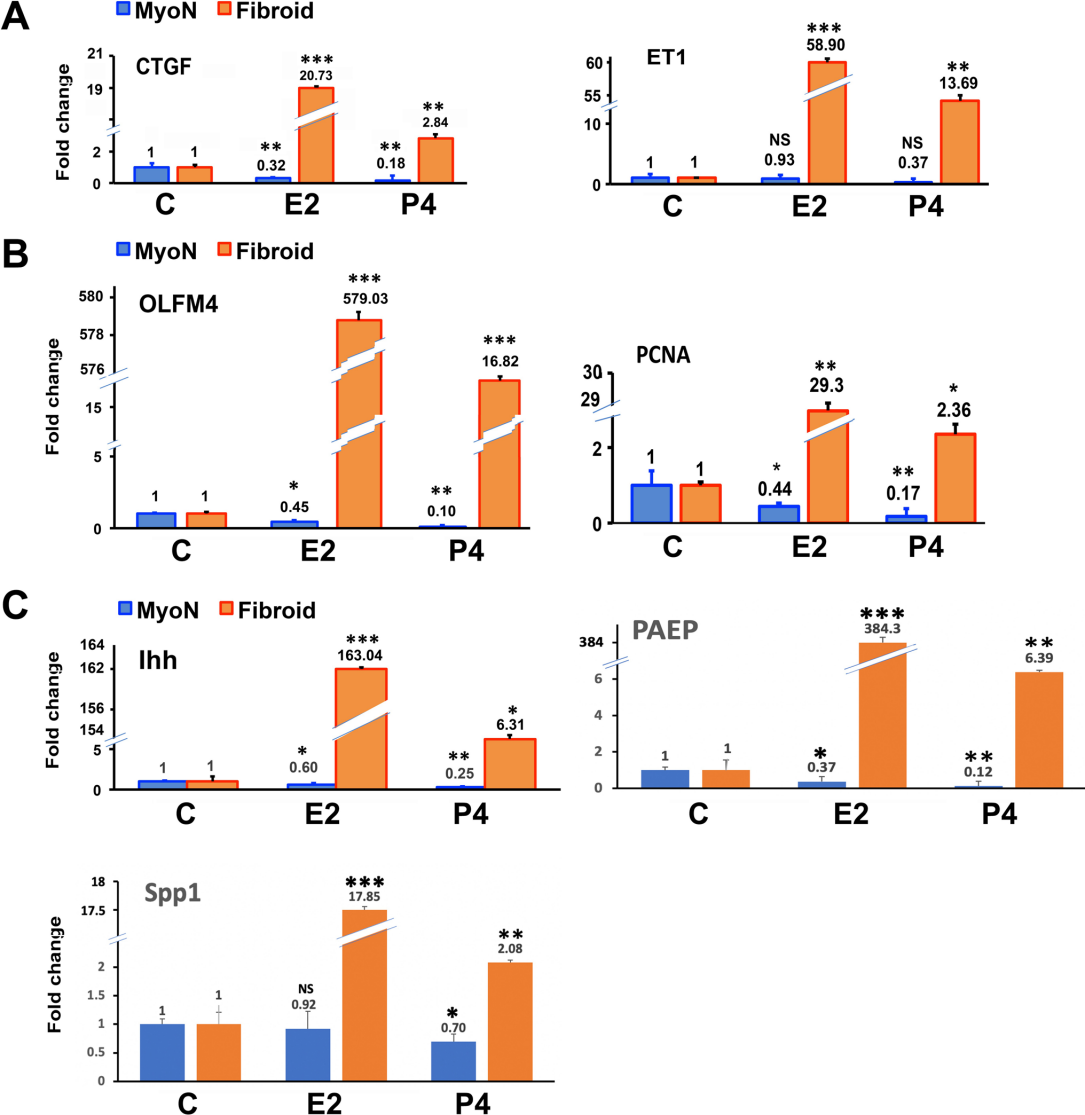
The CD44/Stro1 +  + stem cell-derived UF organoids exhibit hyperresponsiveness with respect to known E2 and P4-stimulated genes when treated with ovarian steroid hormones in vitro. The real-time PCR analysis of Estrogen and progesterone-treated MMSC and UFSC organoids revealed the expression of the proliferation marker *PCNA*, the profibrotic markers *CTGF*, *OLFM-4*, an extracellular matrix protein, *IHH*, a major mediator of progesterone, P4-responsive genes *SPP1,* and *PAEP*. mRNA expressions are expressed in fold change. All bar graphs represent the mean ± SEM of results from five individual experiments (*n* = 5). Asterisks (*) represent unpaired Student’s *t* test, *p ≤ 0.01, **p ≤ 0.001, ***p ≤ 0.0001, NS, not significant

The organoids were harvested to measure the established estrogen- and progesterone-stimulated genes, e.g., *PCNA* (proliferating cell nuclear antigen), *CTGF* (connective tissue growth factor), *ET1* (endothelin 1), *OLFM4* (olfactomedin-4), *IHH* (Indian hedgehog), *SPP1* (secreted phosphoprotein 1), and *PAEP* (progestogen associated endometrial protein) in stem-cell-derived organoids obtained from both normal (MyoN) and fibroid tissues by RT-qPCR. The data are represented as fold changes of E2/P4-treated organoids compared to the untreated samples. As shown in Fig. 4B, the proliferation marker *PCNA* and the profibrotic markers *CTGF* and *ET1* were significantly upregulated in both E2- (10 nG/mL) and P4 (10 nG/mL)-treated UFSC organoids compared to those of MMSC (Fig. 4A, B). The expression of *OLFM*-4, an extracellular matrix protein that is highly expressed in the human endometrium and correlated with the estrogen receptor-α (or estrogen signaling) was upregulated in both E2- and P4-treated UFSC organoids compared to MMSC (Fig. 4B). Even though the *IHH* is known to be a major mediator of progesterone in the uterine epithelium, the expression of *IHH* was significantly upregulated in both E2- and P4-treated UFSC organoids compared to MMSC. The expression of the progesterone-responsive genes *SPP1* and *PAEP* that regulate conceptus implantation and placentation process were also significantly upregulated in P4-treated UFSC-derived organoids compared to MMSC-derived ones (Fig. 4C).

### Increased Extracellular Matrix Protein Is Associated with UF Organoid

**Fig. 5:**
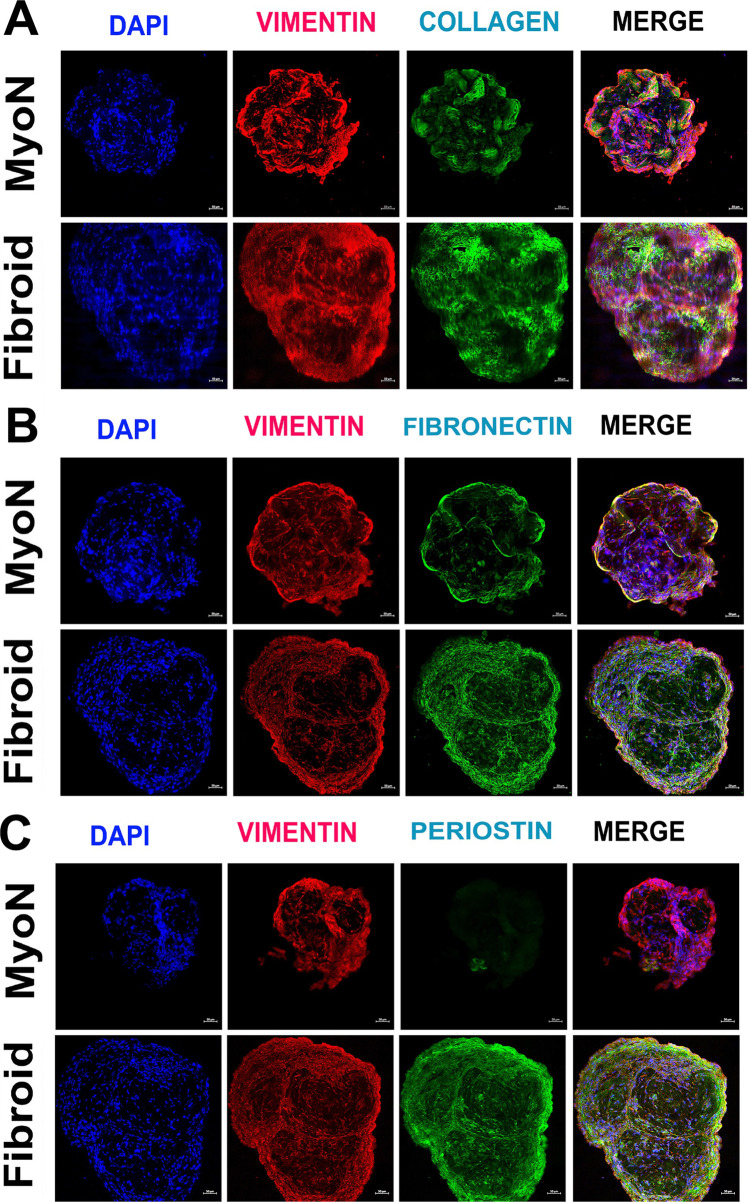
The production of extracellular matrix proteins in human normal myometrial and uterine fibroid (UF) organoids in vitro. The MMSC and UFSC organoids were cryosectioned and processed for immunofluorescence staining for **A** collagen-I (green), **B** fibronectin (green), and **C** periostin (green) and vimentin (red). The nuclei are counterstained in 4′,6-diamidino-2-phenylindole (DAPI, blue). All the data are from five independent (*n* = 5) experiments performed for each individual group

We assessed the production of the extracellular matrix proteins including collagen-I, fibronectin, and periostin by immunocytochemistry of the cryosectioned organoids. The ECM proteins were upregulated in UFSC organoids compared to the MMSC but periostin was significantly higher in UFSC organoids (Fig. 5). Upon treatment with the ovarian steroid hormones, the difference in expression of the ECM proteins was eliminated between MMSC and UFSC organoids (data not shown).

## Discussion

UFs are highly prevalent pelvic tumors in 20–40% women of reproductive age for which new therapeutic interventions are needed [34, 35]. The identity of the factor(s) and molecular mechanisms involved in the cellular transformation of myometrial cells into leiomyoma remains unknown. UFs appear to be estrogen/progesterone-dependent and characterized by excessive production of extracellular matrix [36–38]. Recent findings support the fact that stem cells, growth factors, genetic and epigenetic factors, ovarian steroid hormones, cytokines and chemokines, and ECM components are the key factors involved in the development and growth of UFs [2, 18, 39–42].

Until now, hysterectomy is the only curative treatment for UFs leading to high-cost burden and of public health concern; it is critical to explore the in vitro models that closely mimics the in vivo tumors to understand the pathophysiology of development and disease progression of UFs. Our present study describes the establishment of a stable organoid culture system in vitro starting from human MMSCs and UFSCs derived from normal and UF patients respectively.

In the myometrium, specific cell populations with multipotent stem cell properties have been identified in murine and human models and have been implicated in the plasticity of this organ and in the development of leiomyomas, an unsolved common gynecological pathology [43–47]. Chromosomal and/or genetic alterations are known causes of leiomyoma formation [48, 49], and the mutations affecting mediator complex subunit 12 (*MED12*) were found only in fibroid stem cells [50]. Myometrial stem cells (MMSC) can self-renew and differentiate into new myometrial smooth muscle cells. However, environmental factor-induced genetic/epigenetic abnormalities, as well as ovarian hormone exposure could alter the gene expression pattern and function of undifferentiated myometrial stem cells, leading to the formation of a population of proliferating cells, called tumor-initiating cells, which differ from the rest and could develop into a benign tumor [51]. So, generating a 3D organoid model in vitro from the MMSCs and UFSCs could be a promising tool in evaluating the pathophysiology of the disease progression.

Although conventional pathology describes fibroids as clonally derived from a single clone of smooth muscle cells, it is established that clonal cells differentiate into fibroblast and SMC subpopulation as the fibroid grows [52]. Our study revealed that CD44/Stro1^++^ stem cells isolated from human myometrial (low-risk) and UF (disease) biopsies when grown in two-dimensional culture under normoxic conditions expressed a mixed population of stem cell markers CD44, alpha-smooth muscle actin, the fibroblast cell marker vimentin and ECM marker collagen-I, and fibronectin.

Previous studies have revealed that defects in the DNA damage response network is involved in the development of a variety of diseases. Uterine cells are exposed to various risk factors and mutagenic agents which can cause DNA damage or even affect the DNA repair activities. Epidemiological studies revealed that any defect in the DNA repair activity or accumulation of un-/mis-repaired DNA damages may be involved in UF development and progression [53]. We also observed a downregulation of DNA repair effectors and mediators in CD44/Stro1^++^ UFSCs which are in conformity with the previous UF studies [33]. These results suggest that DNA repair effectors and mediators are impaired in CD44/Stro1^++^ stem cells derived from uterine fibroids revealing the role of dysfunctional DNA repair capacity in increased risk of UF pathogenesis.

The CD44/Stro1^++^ stem cell-derived organoids self-organize to mimic the in vivo structural organization as smooth muscle actin cells organized to the exterior of the organoid with few labeled cellular structures within the interior. Within 7 days, these CD44/Stro1^++^ stem cells-derived organoids recapitulate many of the physiologically relevant properties and features of the in vivo tissue model thus opening the new possibilities to investigate the biological processes involved in disease modeling and testing patient-specific drugs.

The epidemiological, clinical, and experimental evidence support the pivotal role of ovarian steroid hormones in the growth and pathogenesis of uterine fibroids. The effects of estradiol (E2) and progesterone (P4) are interrelated and involve the mediation of receptors, transcription factors, kinase proteins, growth factors, and numerous autocrine and paracrine factors [54]. Previous studies suggested that E2 predominantly increases tissues sensitivity to progesterone by increasing the availability of progesterone receptors, and P4 is required for the complete development and proliferation of UF cells. Still there are conflicting results available about the role of progesterone in UF development, either it is stimulatory or inhibitory [55]. The current study showed that the organoids reproduce the responsiveness to the core regulatory hormone E2 with an overall increase in size but there is a discrepancy between MMSC and UFSC organoids in response to P4. As the vital role of estrogen in uterine growth has been established and leiomyoma growth is closely associated with reproductive years [56], thus, estrogen has received major attention for leiomyoma development. In accordance with the previous studies, we demonstrate that in UFSC organoids, both E2 and P4 upregulate the cell-proliferating activity, whereas in MMSC organoids, without an apparent effect of E2 and P4 as assessed by determining the levels of *PCNA* expression [57]. Real-time qPCR analysis further revealed the specific regulation of several known E2- and P4-responsive markers in the organoid model.

The presence of UFs may interfere with the endometrial pathways involved in the menstrual cycle, leading to heavy menstrual bleeding [58]. Endothelin-1 (*ET1*) is one of the components among other numerous factors involved in spiral artery vasoconstriction and myometrial constriction during the menstruation period [59, 60]. Previous studies also suggested that under the influence of steroid hormones, *ET1* alone or in association with other growth factors could contribute to the complex regulation of uterine tumor growth, such as proliferation, survival, and extracellular matrix production [61]. The expression of *ET1* was significantly upregulated in response to E2 and P4 treatment in UFSC organoids. Similarly, connective tissue growth factor (*CTGF*) has been reported to be associated with a variety of fibrotic disorders which showed a very high expression level especially in E2-treated UFSC-derived organoids [62].

Earlier studies established the fact that cultured UFSC is more responsive to P4 compared to the MMSCs [63]. In this study, we investigated the expression of a few E2 and P4 responsive genes and showed that UFSC organoids are much more responsive to E2 treatment than P4. It is already established that E2 and P4 increase expression of the stemness gene *OLFM4* in the fallopian tube epithelium and is highest in proliferative phase endometrium where E2 stimulates *OLFM4* expression [64]. In accordance with that, treatment with E2 and P4 significantly amplified the *OLFM4* expression in UFSC but the effect of E2 was apparently predominant. Similarly, the expression of Indian hedgehog (*IHH*) which has been shown to be expressed in the uterine epithelium in response to P4 [65] was significantly higher in UFSC organoid in response to both E2 and P4. Furthermore, *IHH* expression was markedly higher in E2-treated UFSC organoids. Similar results were observed when endometrial epithelial organoids of human uterus were treated with estrogen [66]. Therefore, E2 regulation of *IHH* expression in UFSC organoid could be a result of the stem-cell promoting condition of the UFSC organoid culture. 

The growth of UFs is characterized by slow proliferation and is associated with the increased production and concurrent deposition of extracellular matrix proteins usually in a steroid hormone-dependent manner [38]. The extracellular matrix (ECM) comprises most of the tumor growth and consists mainly of glycoproteins, collagens, and peptidoglycans [5, 67]. Some of the abundant and upregulated proteins in fibroids are collagen type-I, fibronectin, and periostin (*POSTN*) [5, 36, 68]. ECM represents the pathological microenvironment which serves as a reservoir for growth factors, cytokines, chemokines, and inflammatory response mediators [3, 69, 70]. The ECM imparts abnormal stiffness to the UF resulting in increased mechanical stress and make it inaccessible to therapeutic agents [71, 72]. Previous studies defined the proteome of uterine fibroids and established that increased *POSTN* production is a hallmark of uterine fibroids regardless of *MED12* mutation status [5]. In compliance with that, we showed that CD44/Stro1^++^ stem cells-derived UF organoids express an elevated level of *POSTN* compared to the MMSC-derived organoids suggesting that UFSC are predisposed to form uterine fibroids.

In this study, we have developed an in vitro sustainable 3D model from CD44/Stro1^++^ MMSC and UFSC acquired from patient samples highly suited to performing functional assays to address many questions about the pathogenesis and treatment of UFs. For instance, the use of 3D cell culture with cell models such as myometrial and UF stem cells would allow greater predictability of efficacy and toxicity before drugs move to preclinical models and clinical trials. Also, studies related to understanding the risk factors associated with the pathogenesis of UFs such as gene X environment interaction and early life environmental exposures to endocrine disrupting compounds (EDCs) will be accessible using the 3D model in a short period of time.

Furthermore, we used RNA and immunohistochemical analysis to study the effect of ovarian steroid hormones E2 and P4 on the organoid size, hormone responsive genes, and ECM protein expression. This analysis revealed that the area size of the CD44/Stro1^++^ UFSC-derived organoids does not change in response to P2 but exhibits a hyperresponsiveness to E2 with respect to the expression of E2 responsive genes. The expression of ECM proteins was upregulated in CD44/Stro1^++^-UFSC derived organoids. Most specifically, the expression of *POSTN* which is a hallmark protein for early-stage leiomyogenesis is significantly upregulated in UFSC-derived organoids. Taken together, this 3D model recapitulates the prominent physiological features of the fibroid and could be useful to develop a new therapeutic intervention to prevent patient-specific UF formation.

## Data Availability

All data and materials as well as software application or custom code support their published claims and comply with field standards.
